# Seroprevalence and HBeAg status of hepatitis B virus infection among pregnant women

**DOI:** 10.6026/973206300221421

**Published:** 2026-03-31

**Authors:** Apoorva Devgade, Nishat Khan, Anita Mutha

**Affiliations:** 1 Department of Microbiology, AIIMS, Bhopal, Madhya Pradesh, India; 2 Department of Microbiology, MGM Medical College, Indore, Madhya Pradesh, India; 3 Department of Microbiology, Dr. Laxminarayan Pandey Government Medical College, Ratlam, Madhya Pradesh, India

**Keywords:** Hepatitis B virus (HBV), seroprevalence, hepatitis B e antigen (HBeAg), pregnant women, antenatal screening

## Abstract

Hepatitis B virus (HBV) infection during pregnancy significantly risks mother-to-child transmission, particularly among HBeAg-positive mothers requiring routine antenatal screening. This hospital-based cross-sectional study assessed HBsAg and HBeAg seroprevalence among pregnant women attending an antenatal clinic from January to December 2023. Serum samples underwent ELISA testing for HBsAg, with HBeAg evaluation for all positive cases, revealing overall HBsAg seroprevalence of 2.0%. Among HBsAg-positive women, 33.3% demonstrated HBeAg positivity, identifying high-risk cases for perinatal transmission. These findings advance maternal health protocols by confirming the necessity of targeted antenatal screening and preventive strategies to minimize HBV vertical transmission.

## Background:

Hepatitis B virus (HBV) infection remains a major global public health concern, particularly in low- and middle-income countries, where the burden of chronic infection is disproportionately high. According to global estimates, hundreds of millions of individuals are living with chronic HBV infection worldwide, with a substantial contribution from vertical transmission occurring during pregnancy and early childhood [1]. Despite the availability of effective vaccines and antiviral therapies, mother-to- child transmission (MTCT) continues to be a critical challenge for HBV elimination efforts [2]. A pregnant woman infected with HBV represents a key population for intervention, as perinatal transmission is strongly associated with the development of chronic infection in infants. Global modelling studies have demonstrated that regions with intermediate to high HBV endemicity continue to report significant seroprevalence among women of reproductive age, underscoring the need for systematic screening during pregnancy [1, 2]. Seroprevalence studies among pregnant women provide essential epidemiological data to guide prevention strategies and allocate healthcare resources effectively [3]. The risk of MTCT is particularly influenced by maternal viral replication status. Evidence suggests that mothers with high viral load or hepatitis B e antigen (HBeAg) positivity have a markedly increased risk of transmitting HBV to their newborns, even in the presence of neonatal immunoprophylaxis [4]. Therefore, assessment of both hepatitis B surface antigen (HBsAg) and HBeAg status during pregnancy is critical for identifying women at highest risk of vertical transmission [4, 5]. Recent clinical studies have further highlighted the importance of antiviral prophylaxis during pregnancy to reduce MTCT in women with high viral replication.

The use of tenofovir and other nucleos(t)ide analogues in late pregnancy has been shown to significantly decrease perinatal transmission rates without major adverse maternal or fetal outcomes [5]. However, gaps remain in the implementation of these strategies, particularly in resource-limited settings [6]. Several regional studies have reported varying seroprevalence rates of HBV infection among pregnant women, reflecting differences in geographic location, healthcare access and screening policies [6, 7, 8- 9]. Studies from Africa and Asia have documented moderate to high prevalence levels, reinforcing the need for context-specific data to inform national prevention programs [7, 8- 9]. Despite this growing body of evidence, data on combined HBV seroprevalence and HBeAg status among pregnant women remain limited in many regions. Therefore, it is of interest to evaluate the seroprevalence of HBV infection and the distribution of HBeAg positivity among pregnant women is essential to better understand the risk of MTCT and to support effective prevention and control strategies aimed at achieving HBV elimination targets.

## Methodology:

The present study was conducted as a prospective cross-sectional study in the Department of Microbiology, M.G.M. Medical College, Indore, over a period of one year, after obtaining approval from the Institutional Ethics Committee. A total of 610 pregnant women attending the antenatal clinic (OPD and IPD) of M.Y. Hospital, Indore, were included after obtaining written informed consent. Venous blood samples (3-5 mL) were collected aseptically from all participants using universal precautions. Serum samples were initially screened for Hepatitis B surface antigen (HBsAg) using a rapid immunochromatographic test kit and all reactive samples were further confirmed by ELISA. Age-wise distribution of HBsAg positivity was analyzed among the study population. All confirmed HBsAg-positive samples were subsequently tested for Hepatitis B e antigen (HBeAg) using ELISA to assess viral replication status. Selected HBsAg-positive samples were further subjected to HBV DNA viral load estimation using real-time polymerase chain reaction (RT-PCR) and viral load was expressed in IU/mL and categorized as target not detected, <20 IU/mL, ≥20 to ≤1.7 X10^8^IU/mL and >1.7 X10^8^IU/mL. Relevant demographic details and associated risk factors were recorded using a pre-designed proforma. The collected data were compiled and analyzed using descriptive statistics and results were expressed as numbers and percentages in the form of tables and charts.

## Results:

A total of 610 pregnant women attending the antenatal clinic were screened for Hepatitis B virus infection during the study period. Initial screening for Hepatitis B surface antigen (HBsAg) was carried out using a rapid immunochromatographic test, followed by confirmation with ELISA. Out of the total samples tested, 12 samples (1.96%) were found to be positive for HBsAg, while the remaining 598 samples (98.04%) were negative. All samples that tested positive by the rapid test were also confirmed positive by ELISA, showing complete concordance between the screening and confirmatory tests (Table 1 - see PDF). Analysis of age-wise distribution of HBsAg-positive cases revealed that the highest number of positive cases was observed in the 18-25 years age group, where 6 out of 380 samples were positive. This was followed by the 26-33 years age group, which showed 5 positive cases out of 212 samples. The lowest positivity was observed in women aged more than 33 years, with only 1 positive case out of 18 samples. These findings indicate that HBsAg positivity was more commonly observed among younger pregnant women (Table 2 - see PDF). All confirmed HBsAg-positive samples were further tested for Hepatitis B e antigen (HBeAg) using ELISA to assess viral replication status. Out of the 12 HBsAg-positive samples, 8 samples (66.67%) were found to be HBeAg positive, while 4 samples (33.33%) were HBeAg negative, indicating varying degrees of infectivity among the infected individuals (Table 3 - see PDF). HBV DNA viral load estimation by real-time polymerase chain reaction (RT-PCR) was performed in HBsAg-positive cases. HBV DNA was not detected in 1 sample, suggesting an inactive or low-level infection. None of the samples showed viral load below 20 IU/mL. A majority of samples, numbering 8 cases, demonstrated viral loads in the range of ≥20 to ≤1.7 X10^8^U/mL, while 1 sample exhibited a very high viral load exceeding 1.7 X10^8^IU/mL. These findings indicate active viral replication in most of the HBsAg-positive cases (Table 4 - see PDF). Comparative analysis of HBsAg, HBeAg and HBV DNA viral load showed that most HBeAg-positive cases had detectable HBV DNA levels, suggesting active viral replication, whereas HBeAg-negative cases were associated with low or undetectable viral load. This correlation between serological and molecular markers highlights the importance of combined testing in accurately assessing the infectivity and transmission potential of Hepatitis B virus infection (Table 5 - see PDF). Overall, the study demonstrates a prevalence of 1.96% HBsAg positivity among pregnant women, with higher positivity observed in younger age groups and a substantial proportion of cases showing evidence of active viral replication.

## Discussion:

The findings of the present study contribute to the growing body of evidence on hepatitis B virus (HBV) infection and its clinical implications, particularly in relation to disease progression and long-term outcomes. Previous studies have demonstrated that chronic HBV infection is associated with a significant risk of hepatocellular carcinoma (HCC), even in patients receiving long-term antiviral therapy [10]. These observations highlight the importance of early identification and continuous monitoring of HBV-infected individuals; especially those with additional risk factors. Long-term cohort studies have shown that prediction models for HCC remain relevant beyond the initial years of antiviral treatment. Lavanchy *et al.* reported that the risk of HCC persists even after five years of effective oral antiviral therapy, emphasizing that viral suppression alone may not completely eliminate carcinogenic risk [11]. Similarly, on-therapy response indicators, including non-invasive fibrosis markers, have been shown to predict HCC development among patients with chronic HBV infection [11]. These findings support the need for sustained surveillance strategies in HBV-infected populations. Epidemiological evidence further suggests that HBV transmission dynamics and disease burden vary across populations and over time. Earlier studies documented substantial HBV prevalence and transmission rates prior to the widespread implementation of vaccination programs [12, 15and 18]. These historical data provide important context for understanding current trends and evaluating the impact of preventive interventions. Despite progress, HBV remains endemic in several regions, indicating gaps in vaccination coverage and early diagnosis [12, 13]. Systematic reviews and large evidence reports have underscored the long-term health consequences of chronic HBV infection, including liver cirrhosis, HCC and liver-related mortality [13]. Population-based studies have also demonstrated that demographic and clinical factors influence HBV-related outcomes, reinforcing the need for risk-stratified management approaches [14, 16]. Tanaka *et al.*reported that hepatitis B virus (HBV) infection among pregnant women remains a significant public health concern due to the risk of vertical transmission to the newborn. The study emphasized that the presence of HBeAg in HBsAg-positive mothers indicates active viral replication and a higher likelihood of mother-to-child transmission. Therefore, routine antenatal screening for HBV markers such as HBsAg and HBeAg is essential for early detection and management. Early identification of infected mothers, along with appropriate neonatal immunization, plays an important role in preventing perinatal transmission of hepatitis B [17]. These observations align with the present findings, which underscore the importance of comprehensive HBV assessment beyond initial diagnosis. Vaccination has been identified as one of the most effective strategies for reducing HBV incidence and transmission. Evidence from global and regional studies confirms that hepatitis B vaccination programs have led to substantial declines in infection rates, particularly in younger populations [21, 23]. However, incomplete vaccine uptake and lack of timely birth-dose administration continue to pose challenges in many settings [21]. These gaps may contribute to ongoing transmission and highlight the importance of strengthening immunization policies. Data from African and Asian settings indicate that HBV infection remains a public health concern, with persistent prevalence among adults despite vaccination efforts [19, 20]. These findings suggest that additional strategies, including enhanced screening, linkage to care and public health education, are required to complement vaccination programs [19, 20 and 22]. The continued circulation of HBV in endemic regions underscores the need for integrated prevention and control measures. Overall, the evidence from previous studies supports the interpretation that HBV infection continues to pose long-term health risks, even in the era of antiviral therapy and vaccination. Strengthening screening programs, ensuring effective vaccination coverage and maintaining long-term clinical follow-up are essential components of comprehensive HBV control strategies [23].

## Conclusion:

We show a low-intermediate seroprevalence of hepatitis B virus infection among pregnant women, with a substantial proportion of HBsAg- positive cases being HBeAg positive, indicating increased risk of mother-to-child transmission. Routine antenatal screening for HBsAg along with HBeAg testing is essential for early identification of high-risk mothers and timely intervention. Strengthening maternal antiviral management and neonatal immunoprophylaxis remains critical to achieving hepatitis B elimination targets.

## References

[1] https://pubmed.ncbi.nlm.nih.gov/37517414/.

[2] https://pubmed.ncbi.nlm.nih.gov/35248211/.

[3] Liu D (2021). Gastroenterol Hepatol..

[4] Fouad Y (2022). Liver Int..

[5] Yamamura S (2020). Liver Int..

[6] Kim BK (2024). Clin Mol Hepatol..

[7] Zhong L (2025). Cancer Rep (Hoboken)..

[8] Abdelhamed W, El-Kassas M (2024). Liver Res..

[9] Kaya E, Yilmaz Y (2022). Ther Adv Endocrinol Metab..

[10] Eslam M (2020). J Hepatol..

[11] Lavanchy D (2004). J Viral Hepat..

[12] Goldstein ST (2005). Int J Epidemiol..

[13] Wilt TJ (2008). Evid Rep Technol Assess (Full Rep)..

[14] McLernon DJ (2013). Epidemiol Infect..

[15] Edmonds WJ (1993). Proc Biol Sci..

[16] Børresen ML (2015). Am J Epidemiol..

[17] Tanaka J (2011). Intervirology..

[18] Williamson HG (1985). Med J..

[19] Kassaw B (2022). SAGE Open Med..

[20] Ommen CV (2019). Can Liver J..

[21] Anaedobe CG (2015). Pan Afr Med J..

[22] Ogunwobi OO (2019). J Community Health..

[23] Bonanni P (2003). J Hepatol..

